# Exploring the usefulness of comprehensive care plans for children with medical complexity (CMC): a qualitative study

**DOI:** 10.1186/1471-2431-13-10

**Published:** 2013-01-19

**Authors:** Sherri Adams, Eyal Cohen, Sanjay Mahant, Jeremy N Friedman, Radha MacCulloch, David B Nicholas

**Affiliations:** 1 Division of Paediatric Medicine, The Hospital for Sick Children, 555 University Avenue, Toronto, ON, M5G 1X8, Canada; 2 Lawrence S. Bloomberg Faculty of Nursing, University of Toronto, 155 College Street, Suite 130, Toronto, Ontario, M5T 1P8, Canada; 3 Department of Paediatrics, University of Toronto, 1 King’s College Circle, Toronto, Ontario, M5S 1A8, Canada; 4 Department of Health Policy, Management and Evaluation, University of Toronto, 155 College Street, Suite 425, Toronto, Ontario, M5T 3M6, Canada; 5 CanChild Center for Childhood Disability Research, 1400 Main Street West, Room 408, Hamilton, Ontario, L8S 1C7, Canada; 6 School of Social Work, McGill, University of Montreal, 3506 University Street, Montreal, Quebec, H3A 2A7, Canada; 7 Faculty of Social Work, University of Calgary, 2500 University Drive NW, Calgary, Alberta, T2N 1N4, Canada

**Keywords:** Complex care, Care plan, Children with medical complexity, Children with special healthcare needs

## Abstract

**Background:**

The Medical Home model recommends that Children with Special Health Care Needs (CSHCN) receive a medical care plan, outlining the child’s major medical issues and care needs to assist with care coordination. While care plans are a primary component of effective care coordination, the creation and maintenance of care plans is time, labor, and cost intensive, and the desired content of the care plan has not been studied. The purpose of this qualitative study was to understand the usefulness and desired content of comprehensive care plans by exploring the perceptions of parents and health care providers (HCPs) of children with medical complexity (CMC).

**Methods:**

This qualitative study utilized in-depth semi-structured interviews and focus groups. HCPs (n = 15) and parents (n = 15) of CMC who had all used a comprehensive care plan were recruited from a tertiary pediatric academic health sciences center. Themes were identified through grounded theory analysis of interview and focus group data.

**Results:**

A multi-dimensional model of perceived care plan usefulness emerged. The model highlights three integral aspects of the care plan: care plan characteristics, activating factors and perceived outcomes of using a care plan. Care plans were perceived as a useful tool that centralized and focused the care of the child. Care plans were reported to flatten the hierarchical relationship between HCPs and parents, resulting in enhanced reciprocal information exchange and strengthened relationships. Participants expressed that a standardized template that is family-centered and includes content relevant to both the medical and social needs of the child is beneficial when integrated into overall care planning and delivery for CMC.

**Conclusions:**

Care plans are perceived to be a useful tool to both health care providers and parents of CMC. These findings inform the utility and development of a comprehensive care plan template as well as a model of how and when to best utilize care plans within family-centered models of care.

## Background

Children with medical complexity (CMC) are vulnerable to care that is inadequate and poorly coordinated [1-6], leading to family stress, unsafe care [3,7-9], poor health outcomes, and increased rates of hospitalization [10,11]. Recent literature strongly advocates that these children be cared for in a medical home [12] and receive a written medical care plan to facilitate their transition through the health care system [12,13]. Work done by Berry and colleagues looking at the experiences of parents and health care providers caring for children with tracheotomy demonstrated the need for provider led care plans and the utilization of web-based technologies in order to enhance care coordination and the secure management of health information across sites of care [14]. Furthermore, the value of care plans in diverse environments and for multiple uses has been established [15-18]. A care plan is a written document that outlines the major medical issues and care needs for a specific child and is created by the health care provider (HCP) in collaboration with the family [19,20]. The document can be modified to meet a variety of needs, for example, emergency care plans, advanced directives, and comprehensive care plans.

Resources and toolkits for the creation of care plans have been developed in recent years [20-22], however, research supporting the content and use of care plans is limited. Care plans have been bundled into some evaluations of the medical home [16,18,23], however, there have been no known studies specifically focused only on care plans. Given that the creation and maintenance of care plans is resource intensive, their value and utility merit focused study.

The aim of this qualitative study was to understand the usefulness and desired content of comprehensive care plans by exploring the perceptions of parents and HCPs of CMC [24,25]. Although all children with special health care needs (CSHCN) may benefit from a care plan, those who are considered medically complex were purposely chosen to study care plans. These children are defined by high health care use, have even greater potential needs for care coordination [5], frequently involving multiple HCPs [26], in various places over long periods of time [27] and thus are likely a data rich patient cohort who would substantially benefit from care plans. An understanding of parent and HCP perceptions in this high-risk population will also have implications for the broader population of CSHCN.

## Methods

### Design

A qualitative approach to inquiry was employed as this iterative, interpretive approach was well-suited to exploring and describing complex and nuanced interactions between parents and HCPs and the subjective experiences of both groups in using a care plan. Specifically, this qualitative study was informed by a grounded theory approach. As an approach to qualitative methodology, grounded theory seeks to generate a theoretical explanation for a specific set of processes or activities that is influenced by a diverse set of perspectives [28,29].

### Setting

Interviews and focus groups with parents and HCPs of CMC were conducted to explore the perceived usefulness and desired content of a care plan at the Hospital for Sick Children, a large tertiary pediatric academic health sciences center, between February 2009 and February 2010. Institutional research ethics approval was obtained prior to study initiation.

### Participants

Participants eligible for recruitment included parents of CMC who had a comprehensive care plan and HCPs who had provided care for a child with a comprehensive care plan. Theoretical sampling, whereby participants are purposively chosen based on emerging themes and theory, was used to guide participant selection.

All eligible parent participants had children who were patients in a complex care program at a tertiary academic health sciences center. The program is a co-management model that has been previously described [30,31]. Inclusion criteria to the complex care program includes children with a chronic condition that are technology dependent and/or users of high intensity care, medically fragile, and require coordinated care due to provision of services by multiple providers in multiple settings. At the time of this study there were approximately 200 patients in this program. All children in this program receive a template-based comprehensive care plan. They do not receive any other forms of care plans. The content and order of presentation included; child’s name, hospital number, insurance information, guardian/parent name and contact information, child’s primary and secondary diagnoses, a brief overview including pertinent emergency medical management, diet, technological supports, a succinct system based review of medical issues with relevant supporting data, a social history section, and contact information for all hospital and community based care providers. The care plan is created by a Pediatric Nurse Practitioner in collaboration with the family. The care plan takes approximately 4–6 hours to create and is integrated into the child’s electronic medical record. The family is also encouraged to carry their own copy and to use it for all health-related interactions [27]. It is updated at clinic visits and during inpatient admissions. Parents recruited for this study were required to have had the care plan for a minimum of 3 months in order to ensure that parents had the opportunity to use the care plan several times and across multiple health care sites. Parent participants were excluded if they could not communicate in English. Children were not invited to participate as the majority were either too young and/or cognitively impaired. Initial purposive sampling for the sample sought sample diversity related to child age, diagnoses, home location, family constellation, and cultural and socioeconomic background. This purposive sampling was augmented by theoretical sampling in the tradition of grounded theory methodology.

Pediatricians and Pediatric Nurse Practitioners within the tertiary academic health sciences center or from related community practices who cared for a child with a comprehensive care plan were invited to participate. Participants were excluded if they had not cared for a CMC with a care plan. Written, informed consent was obtained from all parent and HCP participants prior to their participation in the study. Initial purposive sampling for the sample sought sample diversity related to HCP sub-specialty, inpatient vs. outpatient practice experience and variety of patients cared for. This purposive sampling was augmented by theoretical sampling in the tradition of grounded theory methodology.

### Data collection

Data collection included interviews with parents and focus groups with HCPs. Interviews and focus groups were held concurrently in order to facilitate the constant comparison method of analysis (theoretical sampling and theme saturation). A research assistant (RA) with master level training and extensive experience in focus group facilitation (RM), who was not involved in direct patient care or in construction of the care plan, conducted the focus groups with HCPs. The parent interviews were conducted by two RAs (RM and VJ) with master level training and experience in qualitative interviewing.

Parents of CMC were invited by the study research assistant to participate in an in-depth, semi-structured interview at a time and location of their convenience. Individual interviews afforded parents confidentiality to openly express thoughts and feelings while ensuring flexibility in terms of time and location. The open-ended, semi-structured interview guide was developed iteratively, based on a review of the literature and clinical expertise of the research team. Questions explored parents’ experiences in creating and using the care plan, the meaning of having a care plan, and perceived key components and gaps in the care plan. Interviews lasted between 60 and 90 minutes. Demographic data was obtained from parents including their age, gender, marital status, family income, education level, first language and number of years residing in Canada, as well as their child’s gender, age and number of diagnoses, medications, technologies, emergency department visits, hospital admissions and clinic visits in 2009 (Table 1). HCP participants were invited by the study research assistant to participate in one of three focus groups. This method of data collection was chosen as it generates rich data from all group members within a single meeting and is ideal for participants who will be likely to communicate in a group setting [32] and allows for the rich exchange of ideas among participants.

**Table 1:** Parent, patient, and health care provider descriptive data

**Parent participants*n*=15**	
Mother *n*(%)	13 (87%)
Father *n*(%)	2 (13%)
Age mean(range)	38.5 (20.9-60.4 years)
Marital Status	
Married/Common-law n(%)	11 (73%)
Separated/Divorced/Single n(%)	4 (27%)
Education	
Secondary school	2
Diploma or certificate from trade, technical or vocational school, or business college	1
Diploma or certificate from community college, CEGEP, or nursing school	2
Bachelor or undergraduate degree, or teacher's college	6
Master's degree	4
Personal Income	
No income	2
Less than $5000	1
$5,000-9,999	2
$10,000-14,999	1
$15,000-19,999	1
$20,000-39,999	3
$40,000-59,999	2
$60,000-80,000 or more	3
English first language n(%)	8 (53%)
# of years living in Canada	
5 years or less	2 (13%)
6-10 years	2 (13%)
11-20 years	3 (20%)
20-40 years	2 (13%)
All of life	6 (40%)
**Children/Youth***n*=15	
Sex	
*Female*	5
*Male*	10
Age mean (range)	6.2 (0.8-18.1 years)
# of diagnoses mean (range)	8.6 (5–14)
# of medications mean (range)	9.5 (1–20)
# of technologies mean (range)	2.6 (1–5)
# of ED visits in 2009 mean (range)	3.1 (0–17)
# of hospital admissions in 2009 mean (range)	2.5 (0–8)
# of clinic visits in 2009 mean (range)	17 (4–44)
# of hospital HCPs mean (range)	11.5 (4–18)
HCPs* (*n*=15)	
Role	
Nurse practitioner	5 (33%)
*Respiratory Medicine*	*2*
*Palliative Care*	*1*
*Gastroenterology*	*1*
*General surgery*	*1*
Paediatric Medicine physician	3 (20%)
Community physician	3 (20%)
ER physician	2 (13%)
ICU physician	2 (13%)

*Health Care Provider.

Focus groups were held at the Hospital for Sick Children and lasted 90 minutes. Two focus groups were comprised of four participants and one comprised six participants. Similar to the parent interview guide, the semi-structured, open-ended focus group guide was developed iteratively through review of the literature and clinical experience of the research team. The focus group guide included questions exploring HCP understanding of care plans, their past use of care plans, perceived key components of the care plan and perceived impact of care plans. Information regarding the clinical expertise and experience of focus group participants was also collected (Table 1).

### Data analysis

**Figure 1:**
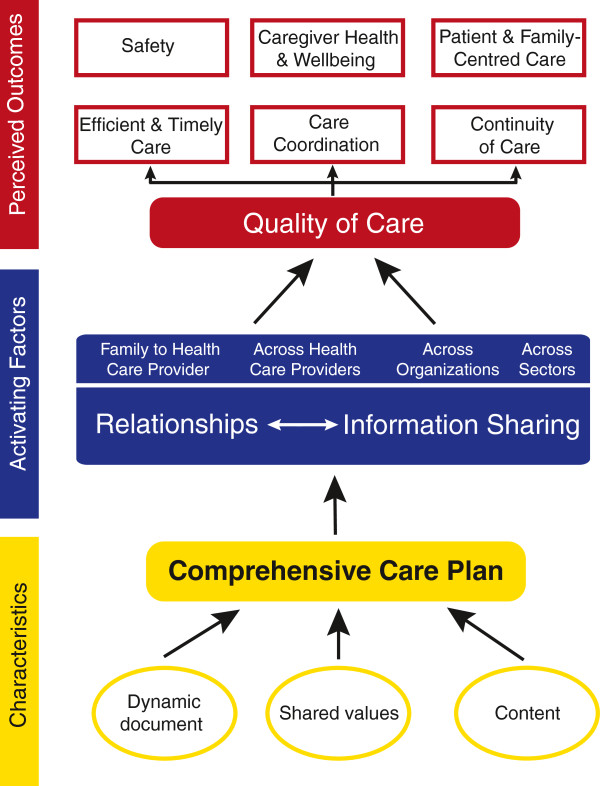
Care plan processes and outcome.

All interviews and focus groups were audio-recorded and transcribed verbatim. Analysis was facilitated by qualitative data analysis software (N-Vivo 8, Massachusetts, USA) [33]. Consistent with grounded theory, data analysis began immediately upon completion of each interview and focus group. This prompt ‘data collection-to-data analysis’ approach, based on tenets of grounded theory methodology, allowed for ‘theoretical’ sampling in formulating and re-formulating working hypotheses, and the efficient development and testing of hypotheses (through subsequent interviews and analysis) and theme saturation [29]. Transcripts were independently coded by three investigators (VJ, SA and RM) and reliability was further ensured by third party review (DN). Analysis comprised three stages: (1) initial categories were created based on review of transcripts (open coding); (2) interconnections between categories were generated (axial coding); and (3) hypotheses regarding the interconnections between categories were proposed (selective coding) [28]. Consensus of codes was achieved through meetings between team members whereby emerging codes, concepts and categories were reviewed and discussed. Finally, a theoretical model was developed to highlight emergent categories and interconnections (Figure 1). Following data analysis, member checking of results with parent participants was completed in accordance with standard qualitative research methodology to ensure the credibility of findings. Member checking occurred through review of the findings by four parent participants, all of whom felt that the findings described their experience. Although member checking was not completed with health care providers, peer debriefing of the findings allowed health care providers to share their insight on the findings. Over a one year period from February 2009 to February 2010 individual interviews (n = 15) were held with parents and three focus groups (n = 15) were held with HCPs of CMC, at which point thematic saturation was felt to be obtained.

## Results

### A theoretical model: characteristics, activating factors and outcomes of care plans

HCPs and parents both identified the care plan as a helpful and needed tool in providing and navigating the complex care of CMC. Participants explained that the care plan described and allowed professionals to respond to their child’s unique health care needs in a comprehensive manner, transcending specific silos of care such as institutions, hospital departments and professional specializations.

Three core components or key themes of the care plan (care plan characteristics, activating factors, and perceived outcomes) emerged through the data to contribute to the formation of a multi-dimensional model of care plan processes and outcomes (Figure 1). *Characteristics* of care plans comprise the dynamic and fluid nature of care plans as they are continually updated with new dynamic content. They are built on a foundation of shared values such as reciprocity, engagement and openness among all in the care circle. *Activating factors* that facilitate effectiveness include key processes such as the enhancement of relational and communication-based components of care between families and HCPs and across HCPs, organizations and sectors. Key perceived *outcomes* were generated, including efficient and timely care, care coordination, continuity of care, safety, patient- and family-centered care, and, ultimately, caregiver health and well-being.

### Characteristics of the care plan

#### Content

Participants identified key components that they felt were essential to the care plan (Table 2). Parents stressed the importance of addressing not only the biological aspects of their child’s health condition but also the social aspects (e.g. supports in home, likes/dislikes of child) within the care plan. A single comprehensive care plan, as opposed to a setting specific care plan (e.g. emergency information forms [17]), was felt to be most useful because it was one document that outlined all of the child’s care needs in an organized and succinct manner and was therefore useful across the entire health care continuum. Parents, who were typically the main ‘carriers’ of the document, felt that it was “complete” and the only document they needed to carry with them,

**Table 2:** Parent desired care plan content

**Parent desired care plan content**	
• Medication list including drug, dose, route, administration instructions	• Brief description of the non-medical characteristics of the child
• List of health care providers and contact information	• The child’s development
	• Pertinent family/social information
• Child’s medical history	• How they communicate
• Emergency care guidelines	• What makes them smile/laugh
• Date and author of the last revision of the care plan	• Things they enjoy
	• Photograph of the child
• Problem/diagnosis list	• Health insurance information
• Alerts section	• Glossary of medical terms
• Allergies	• Summary of technology and/or equipment needs
• Pertinent diet/nutrition information	
• Information about how quickly the child may deteriorate in a crisis	• A summary of the parent’s role/expertise in their child’s care

“It’s really helpful for [the health care team] to get the history, the accuracy of the medications, the dosages, what was done for [my child], and the overall picture of [my child] and not only one side of him, from a hematology point. It’s the whole person here, explained. His whole condition.” – Parent # 6

#### Shared values

The care plan reflected the shared values of HCPs and parents, a characteristic considered to be essential to its creation and ongoing use. A useful care plan was one that was collaboratively created and updated by parents and HCPs on an ongoing basis, with regular opportunities for feedback and revisions as described by one parent,

“It was important to me because up until that point I had felt that no one was listening to me. So I met the nurse practitioner and we developed the care plan. She said ‘come into my office, and we’ll sit down and put a care plan together. And this is going to speak for you.’ And that felt really good because everybody kept saying ‘you’re part of the team’, but I never felt part of the team…” – Parent # 4

#### Dynamic document

The care plan was described by both parents and HCPs as a dynamic document that served as a road map to the child’s care, changing over time according to the child’s changing health care needs,

“[the Care plan is] a comprehensive road map, or Google map, [or Cliff] notes version for complex patients, instead of a thick chart. That summarizes where you’re at, and where you’re going, and who to contact ..” – HCP, Focus Group A, Blue

### Activating factors: how the care plan works

Participants consistently stated that the care plan facilitated positive outcomes including strengthened relationships and enhanced information sharing. The care plan was felt to foster parent empowerment and credibility, clarify parent and HCP roles, and strengthen parent trust and confidence in the child’s care. The care plan was described as a document that “levelled the playing field” between parents and HCPs. It was felt to clarify professional and parent roles and responsibilities of care,

**“**I had this underlying anxiety that they weren’t going to listen to me. Okay, screaming isn’t working, what can I do to have them listen? That’s gone. [The care plan] sort of levels out the playing field. We’re both talking the same language. I may not have medical training, but I have this, I have this piece of paper. It gives me a level of comfort; it gives me a level of security.” – Parent # 4

In times of medical crisis, having the care plan in hand to share with other providers allowed parents to focus on their role and helped other HCPs quickly become oriented to the child’s history and needs,

“I’ve never had a Care Plan before until about a year ago…it’s so crazy coming into the Emerg, and I have to give them all of [CHILD]’s history, his meds, his allergies. Oh my god, it’s so frustrating. And the thing is, every different Doctor you have to do the same thing over and over again…My husband, he’s lost, he can’t do it at all, it’s too much for him…It was so much easier to just hand it over to the Doctors. This is his Care Plan, and I found it saved me a lot of time.” – Parent # 12

The care plan was also felt to enhance health-related information sharing. Participants stated that the plan improved accessibility of care and provided a starting point for discussion about the child’s care, for both HCPs and parents. HCPs expressed how the care plan provided an easily accessible and comprehensive summary of the child’s history, thus facilitating commonplace and difficult discussions with parents. In this respect, the care plan acted as a point of entry to efficient, comprehensive and informed care,

“Instead of taking the history from the beginning, you can confirm things that you see on the care plan with the family. It gives you a starting point for discussion, especially difficult discussions. And if there’s something that you see in the care plan that you’re not sure about, then you can find what you’re looking for in the chart. And it’s much easier because you know what you’re looking for.” – HCP, Focus Group B, Purple

Pertinent health care information was easily and efficiently relayed to HCPs, in turn allowing them to act quickly and confidently. Parents whose first language was not English also expressed that it helped them convey the complexities of their child’s health care needs with more confidence. Moreover, the usefulness of care plans was felt to extend beyond the health care system through enhancing relationships and information sharing across sectors,

“I’ve had the opportunity to collaborate with the complex care team in getting some of these patients home and in the community, and working with them, and caring for these children as they transition into the palliative phases of their illnesses. The Care Plans have been extremely helpful in helping to guide us in what’s happened until this point, and the continuing care, and the advice to our community partners as we help manage them out in the community.” – HCP, Focus Group C, Green

### Perceived quality of care outcomes

Care plans were perceived to contribute to quality of care outcomes (Table 3). These potential outcomes, many of which are consistent with the Institute of Medicine’s domains of quality [34] and/or key constructs of the medical home [35], include enhanced patient safety, caregiver health and well-being, patient- and family-centred care, efficient and timely care, care coordination and continuity of care including timeliness of admission to hospital and medication requisitions.

**Table 3:** Quotations from parents or health care providers (HCPs) related to perceived outcomes

**Theme**	**Quotation**	**Source**
Efficient and timely care	“*It’s so crazy coming into the [Emergency Room], and I have to give them all of [my child’s] history, his meds, his allergies. It’s so frustrating. It was so much easier to just hand it [care plan] over to the doctors. I found it saved me a lot of time.*”	Parent # 12
	“*The medication piece, I think is one of the most imperative in terms of efficiency and reducing error. A lot of these kids are on multiple medications, and usually the care plan is going to be the best place to look for a comprehensive list of medications because it’s incredibly difficult to find elsewhere.*”	HCP*, Focus Group A, Yellow
Safety	*“If you have current, up-to-date information from all people involved, if you have a centralized person who’s communicating with everybody, obviously that’s going to maximize the patient’s care.”*	HCP, Focus Group B, Blue
Caregiver health and well-being	“*It reduces my stress because I feel like there are things I can safely stop trying to keep track of in my memory, without compromising his care. So I also use it as a reference when people are asking me information about his health history. So knowing that I have it as a resource reduces my stress and anxiety.*”	Parent # 3
Patient and family centered care	*There is another benefit I wanted to add. When we are in the hospital and we see doctors for the first time […] they start asking the parents questions. And I can’t tell you how many times I’ve had to give her medical history with all its gory details. And it’s extremely stressful for me. So this [the care plan] saves me from having to deal with that. […] We all go through so much as parents, and we suffer a lot physically, emotionally, psychologically. So this document saves me*.”	Parent # 5
Care coordination	*“It [the care plan] provides a basis or a foundation to begin a discussion. ‘Well, the care plan says this – yes, we will go with the care plan or no, we need to divert from it and this is the reason why’. In its absence, people [health care providers] seem to come at it from a whole bunch of different directions so care seems more fractionated.”*	HCP, Focus Group A, Blue
Continuity of care	*“I do think that it’s [the care plan] one way to enable families to not always feel that they need to be physically in proximity to [a certain] provider. They need to be able to look to other providers and [know] that we can support them to do that.”*	HCP, Focus Group C, Orange

*Health Care Provider.

Parents reported feeling less stressed when they were able to rely on the care plan for information as opposed to having to recount details of their child’s medical history. It acted as a supportive tool as it alleviated the burden of reciting their child’s medical history “one more time”. HCPs identified that the care plan contributed to the continuity of their patient’s care as they were able to work from a document that had been created by one responsible HCP that provided them with the child’s health background and connected them with the child’s larger health care team.

### Areas for development

Participants identified two additional factors important to the successful implementation of care plans: issues related to access (enhancing provider knowledge of the existence of care plans and ensuring providers have timely access) and issues related to the currency and accuracy of care plans. The administration of the care plan emerged as a key issue related to the effectiveness of care plans. Specifically, how the care plan was shared, accessed and updated between HCPs and parents was directly related to the usefulness of the care plan and noted as key areas for improvement. Participants suggested standardizing the administration of the care plan and called for enhanced support for and awareness of the care plan across health care institutions. Parents and HCPs shared thoughts about how best to achieve this aim, including: (1) storing the care plan on a centralized electronic database accessible by all involved HCPs; (2) initiating regular, scheduled reviews of the care plan by the parents in collaboration with one designated HCP; and (3) providing education about the implementation and use of the care plan itself to HCPs and institutions. Work in this area suggests the utilization of new technologies (e.g. internet for shared care plans) however barriers related to privacy and personal health information continue to slow progress [14]. There was discordance between parents and HCPs as to who was viewed as the best person to update the care plan. Some parents expressed the desire to be able to update the care plan themselves while some HCPs felt this would decrease the credibility of the document. Parents also expressed that the care plan was still the most trustworthy document (regardless of the date of last revision) while HCPs had difficulty relying on the care plan if it had not been updated recently. Participants suggested standardizing the administration of the care plan. Those who did not regularly use care plans reported that their successful implementation was hindered by administrative challenges of accessing and sharing the care plan across HCPs and institutions. This mirrored some of the greater systemic challenges inherent in the current health care system for children with complex health care needs.

## Discussion

Emerging research demonstrates the benefits of care plan use for children with complex health care needs [14,16,18]. While much of this research has examined the usefulness of care plans in conjunction with formal care coordination programs, to our knowledge there is a dearth of research that has explored parent perceptions regarding the content of a care plan or its usefulness as a tool independent of a program of care. Almost universally, the care plan was viewed as a useful tool for both HCPs and parents. Most notably, the care plan emerged as a tool that centralized and focused the care of the child and levelled the hierarchical relationship between HCPs and parents, thereby enhancing the reciprocal exchange of information and strengthening relationships. Parents described how the care plan enhanced their feelings of empowerment and credibility. The care plan was described as a collaborative, comprehensive document that acknowledged parents as experts and advocates in their child’s care. Interestingly, in their study examining the perspectives of parents and physicians on the role of parents as information intermediaries, Stille and colleagues found that parents were more comfortable than physicians in holding this role. However, the degree to which parents felt comfortable in this role varied [27]. These findings reveal an important implication of the findings from this study in that care plans should be considered a complementary practice. From a safety lens the content of the care plan has the potential for varied use and interpretation which could equally result in a positive or negative outcome. The care plan should not replace discussions between parents and HCPs related to the child’s most up-to-date treatment plan and it should not be assumed that all parents are similarly comfortable acting as information intermediaries. Furthermore, clear guidelines around who updates the care plan and how often are crucial.

Care plans are used nationally and internationally and have been described in many different forms – from formal, clinician-developed comprehensive care plans to informal, parent-developed summaries of a child’s health [20,21,23,36]. This exploratory study offers positive preliminary findings that suggest that comprehensive care plans are a useful component of care for CMC, complementing emerging research in this area. One study of a Medical Home [36] briefly mentioned using care plans as part of a range of interventions aimed at improving care coordination for children with chronic conditions [37]. These authors reported family satisfaction with use of the care plan specifically in helping explain a child’s condition and medical issues to school and emergency department personnel. Our findings are consistent with earlier reports and research on broader issues of care provision for CMC which emphasize the association of parent-professional partnerships and enhanced communication and information sharing, with improved outcomes for CSHCN and their families [38,39].

Several limitations of this study are noted. First, as is common with qualitative research, interview and focus group discussions reflected the experiences of a limited number of participants and in this case, all from one tertiary care centre. This study occurred in Ontario, Canada where there is universal access to health care. This may limit the generalizability of findings to other jurisdictions which might have fewer or greater barriers, depending on the structure of health records, the system of support of families, and the accountability and responsibilities of care providers.

Efforts were made to capture a range of experiences with the care plan and sample variation, and discussion with all participants revealed many common and/or shared experiences. A minimum 3 month usage time was used for care plan study eligibility, but in doing so, despite actively seeking out negative cases, some informative cases in which care plans were not found to be helpful may have been missed. There was under-representation of primary care paediatricians in the focus groups compared with broad populations of CSHCN cared for in a medical home. However, as this sample of CMC were often acutely unwell and received care under a hospital-based medical home, which is a model of care delivery for this population that has grown exponentially in prevalence in the last few years, participating HCPs represented the typical circle of care for these families [40]. Furthermore, while participants represented a culturally diverse sample, only participants who could communicate in English were included. CMC were purposely chosen to study care plans. These children are defined by high health care use, frequently involving multiple HCPs, in various places over long periods of time, and thus are likely a patient cohort who would substantially benefit from care plans. Although findings appear to be applicable to the broader group of CSHCN it is not know if less complex sub-groups of CSHCN would demonstrate similar findings. Further, the relationships in the emergent model are hypothesis generating; hence, research using quantitative methods to examine outcomes of care plan utilization is needed.

**Figure 2:**
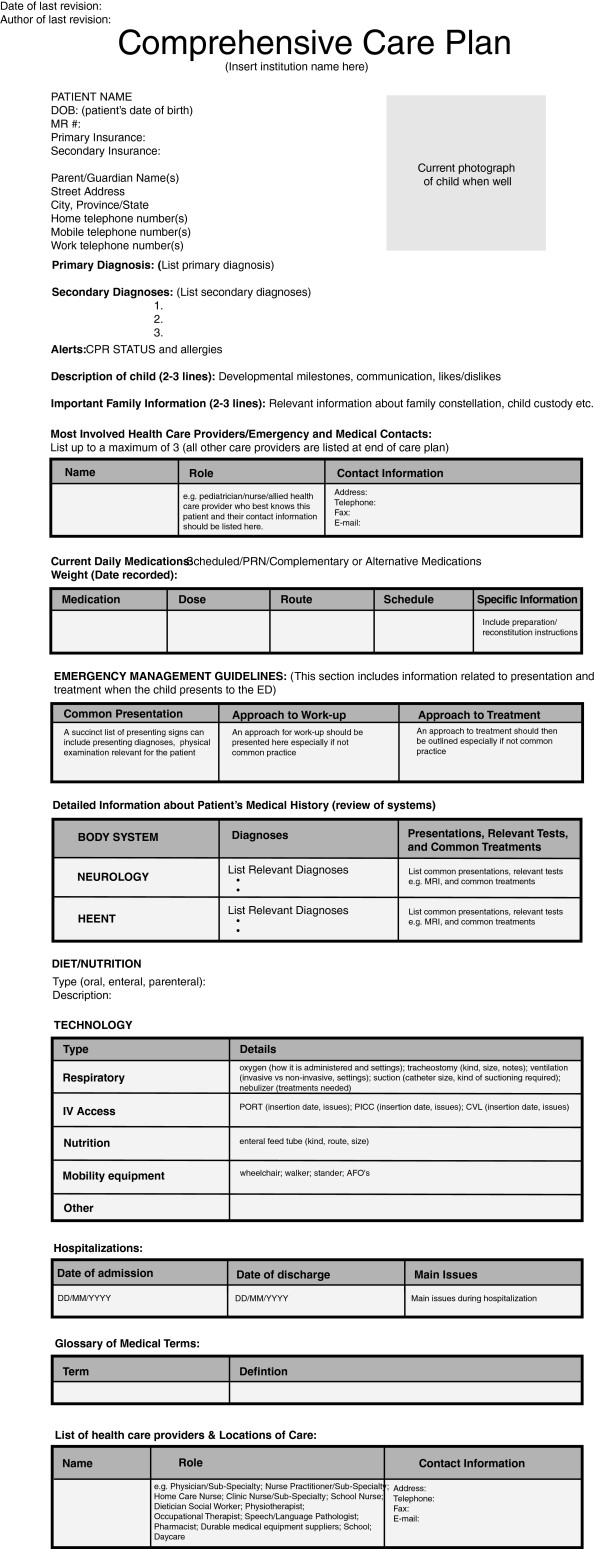
Care plan template.

The findings from this study have implications for the design and implementation of care plans for CMC. The findings support a care plan template that is family-centered and includes content relevant to the medical and psychosocial needs of the child. A modified comprehensive care plan template based on participant feedback can be seen in Figure 2, however, systematic evaluation is recommended prior to implementation. An integrated information system created for the development and sharing of care plans across providers and settings is essential. This can help facilitate accessing accurate information in a timely manner. An important contributor to the success of care plans is uptake by HCPs. As stated by many parents, the care plan is only useful if the HCP takes the time to read it and utilize it. The promising application of contemporary technologies such as mobile devices integrated into existing electronic health records may enhance the accessibility of care plans for ease of reading. Research addressing the further development and validation of comprehensive care plans and their impact on perceived outcomes identified in this study is needed in ultimately providing outcome data, examining optimal strategies for implementation, and costs associated with care plan creation and maintenance.

## Conclusions

Parents and HCPs perceive a care plan to be a useful tool in the care of CSHCN with medical complexity. By strengthening relationships and enhancing information sharing, care plans are perceived to improve quality of care in multiple domains. Further study to examine the effectiveness of care plans and best practices for implementation has the potential to further improve the quality of care delivered to these children.

## Abbreviations

CSHCN: Children with special health care needs; CMC: Children with Medical Complexity; HCP: Health Care Provider.

## Competing interests

The authors declare that they have no competing interests.

## Authors’ contributions

SA conceived of the study, acquired funding, participated in study design and coordination, participated in data analysis and interpretation and drafted the manuscript; EC participated in the study conception and design and helped to draft the manuscript. SM participated in data analysis and interpretation and helped to draft the manuscript. JF participated in study design and helped to draft the manuscript, RM participated in data collection, analysis and interpretation and in manuscript preparation, DN participated in the design of the study, oversaw the qualitative analysis, participated in qualitative data analysis, and helped to draft the manuscript. All authors read and approved the final manuscript.

## Pre-publication history

The pre-publication history for this paper can be accessed here:

http://www.biomedcentral.com/1471-2431/13/10/prepub
